# Molecular sexing in Japanese murrelet (*Synthliboramphus wumizusume*) and a tandem-repeat polymorphism on the W chromosome

**DOI:** 10.1038/s41598-020-65206-7

**Published:** 2020-05-22

**Authors:** Hitoshi Hatakeyama, Yutaka Nakamura, Takahiro Konaka, Shin Nishida, Wannapimol Kriangwanich, Kazuyoshi Uematsu, Shuichi Tsuchida

**Affiliations:** 1 Laboratory of Comparative Cellular Biology, Nippon Veterinary and Life Science University, 1-7-1, Kyonan-cho, Musashino, 180-8602 Tokyo Japan; 2 Miyazaki Wildlife Research Group, 1-5-14, Gakuenkibanadai-kita, Miyazaki, 889-2152 Japan; 3 Faculty of Education, University of Miyazaki, 1-1 Gakuen-Kibanadai-Nishi, Miyazaki, 889-2192 Japan; 4 Department of Veterinary Bioscience and Public Health, Faculty of Veterinary Medicine, Chiang Mai University, Chiang Mai, 50100 Thailand; 5 Natural Resource Damage Assessment of Asia, 363-3 Ajiro Akiruno, Tokyo, 190-0155 Japan

**Keywords:** Conservation biology, Comparative genomics

## Abstract

The Japanese murrelet (*Synthliboramphus wumizusume*) is an endangered small seabird species in Japan. Molecular sexing using PCR targeting of the gene encoding *chromodomain helicase DNA-binding protein 1*(*CHD1*) has been used for sex identification. Specifically, PCR using any of three commonly used primer sets (CHD1F/1R, 2550F/2718R and P2/P8) has permitted sexing in many bird species. CHD1F/1R and 2550F/2718R permitted molecular sexing in Japanese murrelet; however, P2/P8 did not permit. To generate a primer pair that permits efficient molecular sexing in this species, a new primer set, CHD1F1/1R1, was prepared to permit amplification of smaller products from degraded DNA samples. The electrophoretic patterns of PCR products amplified with the new primer set were easily classified as female or male. Additionally, the PCR product indicated the presence of a polymorphism in the fragment from chromosome W. The PCR fragments of long-type (WL) and short-type (WS) polymorphisms were observed only in females. When the distribution of the *CHD1* gene on chromosome W of 61 female Japanese murrelet on Biroujima Island in Miyazaki Prefecture, WL and WS were observed in 90.2% and 9.8%. The DNA polymorphism is derived from the number of copies of a 32-bp-repeat unit, with WL and WS corresponding to two and one 32-bp-repeats, respectively.

## Introduction

Japanese murrelet (*Synthliboramphus wumizusume*) is a small bird that has been classified as a member of the Alcidae family. This bird inhabits and breeds on inhabited areas of islands in temperate waters around Japan^1–4^ and South Korea^5^. The total population of Japanese murrelet is estimated to be approximately 4,000–10,000 individuals^1^. The species is the rarest Alcid in the world and has been designated as having Vulnerable status on the IUCN Red List of Threatened Species and as a vulnerable Species Class-II by the Minister of the Environment of Japan (Red List, Ministry of the Environment, 2019). In the Japanese murrelet, as in approximately 20–30% of known bird species, females and males have very similar external morphologies, and the two sexes cannot be distinguished based on body weight or behavior^6^. A survey of the external morphology of the Japanese murrelet has shown that males and females are very similar in size, body weight, coat color, and behavior in all life stages; even in the later breeding period, when the body weight of the females decreases significantly, that of males also decrease in the same way. Thus, it is very difficult to distinguish between male and female Japanese murrelets by observation of external morphological characteristics^2^. Unambiguous identification of the sex of each animal is an essential process in the research of many biological fields, especially that related to ecology, behavior, and conservation studies.

PCR-based methods have been applied widely to molecular sexing for many kinds of birds, providing rapid and accurate results^7–10^. All bird species employ two sex chromosomes, Z and W. Male birds are homogametic(ZZ) and female birds are heterogametic(ZW). Constituent differences in the sex chromosomes between males and females therefore can be applied to DNA-based identification for sexing of birds. Notably, the highly conserved *Chromodomain helicase DNA-binding protein 1(CHD1)* gene is present in single copy on Z and W, but the alleles typically differ in intron lengths. When the distinct intronic regions within the *CHD1* genes are amplified using a specific primer set corresponding to the highly conserved exon regions, the amplicons generated from the two sexes yield distinct band patterns on agarose gels. Specifically, females typically exhibit two bands of different lengths, one from chromosome Z and the other from chromosome W. In contrast, males typically exhibit a single band that contain two copies of the identically sized product from chromosome Z^8,9,11,12^. When the amplified intronic regions from chromosomes Z and W are similar and overlay each other on electrophoretic gels, some treatment is required to distinguish the DNA sequences of the amplicons.

In the present study, we focused on sexing of the Japanese murrelet. A species-specific primer set was prepared for sexing of the Japanese murrelet and compared to the widely used, popular primer sets that have been used for sexing in birds. Notably, the DNA fragments amplified with the new primer set revealed a polymorphism within this species. This primer set is expected to be useful not only for molecular sexing of the Japanese murrelet, but also for distinguishing the females into two groups defined by this polymorphism. We showed that this polymorphism is a new DNA marker that corresponds to a variable number of tandem 32-bp-units on chromosome W in the Japanese murrelet.

## Results

### Application of three previously reported, popular CHD1-targeting primer sets for molecular sexing of the Japanese murrelet, and preparation of a new Japanese murrelet species-specific primer set

The three frequently used *CHD1*-targeting primer sets were applied for molecular sexing in the Japanese murrelet. Two of these three primer sets, 2250 F/2718 R and CDH1F/CDH1R, produced electrophoretic patterns similar to those reported previously in many species of birds. Specifically, males yielded a single band, while females yielded two bands, one of which matched the size of the male-specific band. The shared band presumably corresponded to a *CHD1* homolog on chromosome Z, while the second band appeared to correspond to the *CHD1*gene on chromosome W. In contrast, the P2/P8 primer set yielded a single band from all animals; presumably this band corresponded to a mix of *CHD1Z* and *CHD1W* amplicons running at the same size or this primer set did not permit amplification from chromosome W. However, comparison of the electrophoretic patterns indicated there were noteworthy differences in the sizes of the products. When molecular sexing is carried out using non-invasive samples from wild birds, smaller-sized fragments seem to be amplified more efficiently than are larger-sized fragments. Therefore, we designed a new Japanese murrelet-specific primer pair predicted to yield a smaller PCR product from chromosomes Z and W than were obtained from the previously described primer sets. Indeed, the fragments amplified with the newly designed primer set were smaller in size and more efficiently amplified than those obtained with the previously reported, popular primer sets 2250F/2718R and CDH1F/CDH1R.

### Estimation of the utility of the species-specific primer set for Japanese murrelet

Because of many restrictions on the capture of specific birds and on the collection of invasive samples, it was not easy to obtain enough samples as controls. Furthermore, the Japanese murrelet is a monomorphic species, so it is difficult to distinguish female from male based solely on external morphological survey; this point means that it is hard to rapidly and accurately determine animal sex in the field. Therefore, the species-specific primer set CHD1F1/CHD1R1, which was generated as part of the present study, was evaluated for its utility in accurate molecular determination of sex in the Japanese murrelet based on the following reasoning. First, comparative tests were performed using three of the primer sets described above (CHD1F/CHD1R, 2250F/2718R and CHD1F1/CHD1R1) along with the DNA samples extracted from blood clots of Japanese murrelet selected at random, without prior information about animal sex. The results obtained with each primer set were found to be in complete agreement with each other. Second, the newly designed primers for the Japanese murrelet corresponded to DNA sequences homologous to *CHD* coding regions highly conserved among many avian species. The highly homologous regions in the primers were expected to make it possible to use this primer set for molecular sexing in a variety of bird species. As a practical example, the primer set was applied to another bird species, Japanese quail, which presents sexual dimorphism as adults and in which sex is readily identified based on external morphological features. Within the Japanese quail, there was no discrepancy between molecular sexing with the species-specific primer set and sexing by traditional methods based on morphology (Supplementary Fig. 2). Third, one Japanese murrelet was found dead in Biroudo island, permitting direct determination of sex by inspection of reproductive organs at necropsy. In parallel, a DNA sample was extracted from the feathers of this bird, permitting correlation of the sex as determined by molecular sexing with the CHD1F1/CHD1R1 primer set, and sex as determined by inspection at necropsy. Although there were not additional Japanese murrelet samples for which sex was known both by molecular sexing and by anatomical structures, the existing samples worked well as a positive control, permitting a distinction between results from males and females. Therefore, we conclude that the new primer set provides accurate results when used for sexing of the Japanese murrelet.

### A variable number of tandem repeats serve as a DNA marker on chromosome W of Japanese murrelet

**Figure 1:**
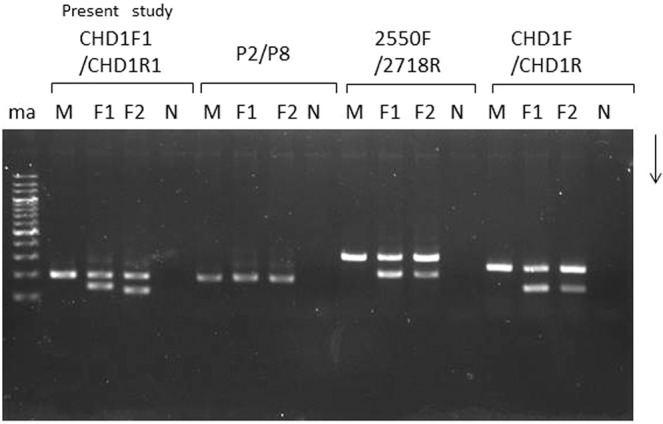
Comparison of agarose gel electrophoretic patterns of the PCR products amplified by each of the four primer set tested for sexing of Japanese murrelets. CHD1F1/CHD1R1 was designed in the present study; P2/P8, 2550F/2718R, CHD1F/CHD1R were described previously. For each primer set, three samples (consisting of one male (M) and two female(F1 and F2) Japanese murrelets were analyzed. N indicates the negative controls (reactions performed without sample DNA). With the exception of P2/P8, all primer sets were able to distinguish the chromosome Z-derived fragments from the smaller chromosome W-derived fragments. The ma line indicates the 200-bp DNA ladeder for a marker DNA.

**Figure 2:**
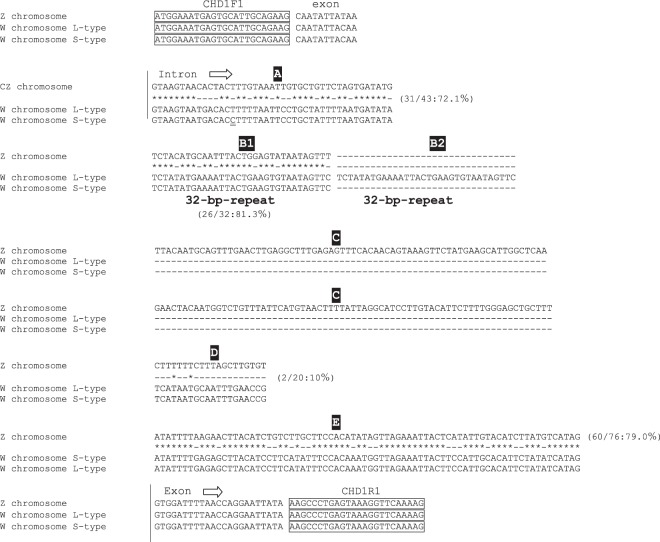
Alignment of three DNA sequences of Z chromosome (Z/Z male), W chromosome L-type (clone WL) and W chromosome S-type(clone WS), which are GHD1-related PCR products including the region of the parts of two exons and the intron between the exons. Dash(–) indicates the delete of nucleotide. The intron starts at GT and finished at AG, this sequence agrees with GT-AG rule. The boxed DNA sequences indicate the primer sequences. Comparison of DNA sequences among the PCR products for chromosome Z, WL and WS make it possible to divide the intron region into 5 regions, from A to E. B1 and B2 indicate the polymorphic region. Comparison of the DNA sequences of chromosome W clones indicates that the polymorphism in chromosome W is derived from the number differences of a 32-bp-repeat. When the DNA sequences of chromosome Z and W are compared each other, the region A and E show highly homologous. On the other hands, D shows a striking difference. Region C is observed in only chromosome Z.

**Figure 3:**
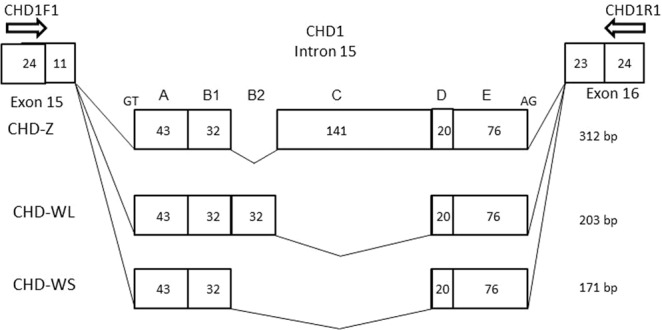
Schematic figures of the PCR products from chromosome Z and two polymorphic clones from chromosome W, WL and WS clones. The intronic regions origin at GT and end at AG as in GT-AG rule of introns. The estimated length of introns in CHD-Z, CHD-WL and CHD-WS, amplified with the primers made in this study, CHD1-F1/R1, were 312, 203 and 171 bp, respectively. Polymorphic region of the intron of *GHD1* genes on chromosome Z and W of Japanese murrelet. The region containing a polymorphic intron divided into five areas, A to E. Comparison between CHD-WL and CHD-WS indicated that the DNA sequences of this region were coincided perfectly with two exception with the copy number of a-32 repeat and one nucleotide change in region A. On the other hand, comparison between CHD-Z and CHD-W, the region C exites in only CHD-Z. The regions of A and E have more than 70% homology, however, B involved repeat region and D region in a-32-bp-repeat have lower homology than regions of A and E.

When *CHD*-based molecular sexing was carried out using the species-specific primer set, CHD1F1/CHD1R1, and the products were separated by agarose gel electrophoresis, two different patterns were observed in female samples (Fig. 1, Supplementary Figs. 1 and 2), in which the patterns were clearly differentiated by the difference of space in width between the common (Z-derived) band shared between males and females and two patterns obtained from female Japanese murrelets reflect a polymorphism within this species. As noted above, agarose gel electrophoresis of the products from males and females yielded distinct patterns: two bands were observed in females compared to one in males. The upper fragment was shared in common by both male and female samples and therefore was presumed to be derived from the *CHD-Z* allele. Additionally, a female-specific (presumably W-derived) band was detected. However, female Japanese murrelets yielded either of two different sizes of W-derived amplicons, defined as long-type or short-type, on agarose gel electrophoresis (Fig. 1, F1 and F2; Supplementary Fig. 1, ZWL and ZWS; Supplementary Fig. 2, Lanes 5 and 6). PCR products from female short-type (WS) and long-type (WL) of chromosome W were cloned into T-vector and analyzed by polyacrylamide gel electrophoresis (Supplementary Fig. 1). The resulting fragments were subjected to sequencing (Fig. 2); an alignment between the sequences is shown schematically in Fig. 3 (MN519214 from CHD1Z gene and MN519215 from CHD1W gene). Alignment of the two different DNA sequences indicated that the polymorphism observed in chromosome W is derived from variation in the number of 32-bp-repeat units. Specifically, WL has two repeat units. The DNA sequence alignments also revealed that each of the three PCR products obtained from the Japanese murrelet using the CHD1-F1/R1 primer set consist of a *CHD* intron flanked by pieces of the two adjacent exons. The intron starts with a GT and finishes with an AG, which appears to be in accordance with the rule for exon-intron junctions. The lengths of the DNA fragments of the intronic regions for the Z, WL, and WS-derived fragments are 312 bp, 203 bp and 171 bp, respectively.

The new primer set, CHD1-F1/R1, prepared in the present study is expected to be useful for molecular sexing in the Japanese murrelet, and especially using degraded DNA samples. Additionally, the results of agarose gel electrophoresis simultaneously classified the females into two groups based on the presence of a chromosome-W polymorphism. Distribution of a 32-bp-repeat polymorphism in the intron of the *CHD1* gene on chromosome W of the Japanese murrelet on Biroujima Island in Miyazaki Prefecture in Japan is shown in Table 1. In the 61 females identified among 149 individuals, 9.8% of the females harbored 1 copy of the 32-bp-repeat polymorphism. The remaining 90.2% of the females harbored 2 copies of the 32-bp-repeat polymorphism.

**Table 1:** Distribution of 32-bp-repeat polymorphism in the intron of the *CHD1* gene on chromosome W of Japanese murrelet inhabiting Biroujima island, Miyazaki Prefecture in Japan.

Sex	No.	%	WL or WS	No.	%
♂	88	59.1 %			
**♀**	61	40.9 %	Long	55	90.2 %
			Short	6	9.8 %
Total	149	100.0%		61	100.0%

Females were classified into two types. Long-type female has two of tandem repeats of 32-bp-units and short type has one unit.

## Discussion

The Japanese murrelet inhabits and breeds on the islands around Japan. Although sex identification is very important for biological and ecological field studies, it is difficult to distinguish males and females of this species by the traditional method of inspecting external features, given that the species exhibits monomorphic characteristics. In other species, molecular sexing using PCR methods targeting the *CHD1* gene has made it possible to identify the sex rapidly and accurately using non-invasive samples^6,7,13,14^. Japanese murrelets are morphologically monomorphic birds, meaning that it is hard to distinguish between males and females. Molecular sexing is an attractive method for sex identification. Typically, the PCR products should be smaller than 300 bp to permit potential application to noninvasive samples^15^. Use of two of the standard primer sets, 2250F/2718R and CDH1F/CDH1R, with Japanese murrelet DNA produced larger fragments, exceeding 500 bp in size (Fig. 1). Smaller fragments are amplified more efficiently than larger regions, especially, when amplifying from highly degraded DNA extracted from noninvasive samples^9,16,17^. Although the utility of primer sets including newly and commonly used primer set was not estimated directly, these primers will be useful for many kinds of bird species. The new primer set described in the present work was designated to amplify smaller fragments that were still large enough to visualize by routine agarose gel electrophoresis with ethidium bromide staining and UV detection. The Japanese murrelet is a vulnerable wild animal and the capture is restricted. The specimens used to extract DNA often are obtained by non-invasive methods, and the extracted DNA might be low in quantity and quality (highly degraded). As we showed here, a primer set targeting a smaller amplicon of a potential sex chromosome-linked marker, the *CHD1* gene, is able to determine the sex of Japanese murrelet reliably. The PCR products amplified with the improved species-specific primer set, CHD1-F1/R1, revealed not only the sex by molecular sexing (using the *CHD1* genes), but also permitted classification (via routine agarose gel electrophoresis) of females into either of two types based on a polymorphic DNA marker in the intron of the *CHD1* gene on chromosome W. The polymorphism corresponds to the variable copy number of a 32-bp-tandem-repeat in the gene on chromosome W.

A newly designed species-specific primer set was prepared in this study for molecular sexing in Japanese murrelet. The PCR products amplified with the primer set make it possible to identify the sexes, while simultaneously classifying females into two groups. By molecular sexing, the agarose gel pattern of PCR products showed one and two bands in male and female, respectively. The single band obtained in males corresponded to amplicons of the same size generated from the copies of *CHD1* on each of the Z chromosomes; the two bands obtained in females corresponded to separate amplicons generated from separate copies of *CHD1* on chromosomes Z and W. Additionally, we detected two types of W bands, long and short. Cloning and sequencing of amplicons from the two types indicated that the presence of a variable copy number of a 32-bp-tandem repeat is the cause of the polymorphism. Chromosome W-short-type (WS) has one repeat and W-long-type (WL) has two repeats. This polymorphism in the PCR products is presented schematically in Fig. 3. Comparison of the alignments between two W types of PCR products, WL and WS, shows that the 32-bp-repeat is in complete accord, although a single nucleotide substitution was observed in the DNA sequence before the 32-bp-repeat region. The comparison between the PCR products derived from chromosome Z and chromosome W suggested that the regions with highly conserved DNA sequences are observed not only in the exons, but also in some regions of introns. Homology of exons and introns indicates that the *CHD1* genes on chromosome Z and W were the same in the genetic past. Comparison of the locations of the 32-bp-unit sequence among various species suggests that the repeat is located within intron 15 (between exons 15 and 16) within the *CHD1* gene on chromosome W. BLAST analysis was carried out in an attempt to determine the origin of the 32-bp-unit. This analysis (data not shown) indicated that more than ten avian species have identity for the 32-bp-unit, while many additional bird species have highly similar sequences units with some substitutions. Copies of the 32-bp-repeat-units were detected widely in bird genomic DNA. Previous work described polymorphic size differences on chromosome Z^18^, but did not identify variable tandem repeats. Thus, the polymorphism detected in Japanese murrelet was characteristic of the structure of 32-bp-repeat unit and the location on sex chromosome W. there is no functional information related to the polymorphism. However, polymorphism is a useful marker to separate the chromosomes. The accumulation of DNA polymorphisms are expected to lay the foundation of the study in future.

In conclusion, accurate sex identification is necessary to manage and conserve endangered wild birds, especially monomorphic birds like the Japanese murrelet, in which males and females cannot be distinguished based on external features. Molecular sexing makes it possible to identify the sexes by PCR methods, which will in turn facilitate determination of the structure of the sex chromosomes. Accurate and distinct identification for sexing depends on the primers used for the PCR reactions. In the present study, we presented a new primer set that permits molecular sexing from degraded DNA samples while simultaneously permitting classification of the females into two groups based on a copy-number polymorphism representing a variable number of tandem repeats.

## Methods

### Ethical approval

This study was carried out in accordance with the Act on Welfare and Management of Animals (Law No. 105, Japan) and the guidelines published by the Nippon Veterinary and Life Science University (https:www.nvlu.ac.jp/research/ani-exp.html/). All experimental protocols were approved by the Animal Care and Use Committee of Animal Ethics of the Nippon Veterinary and Life Science University (permission number #2019S-23). To capture the seabirds and collect the blood samples are authorized and managed with the permissions from the Ministry of the Environment (#10-0158), Miyazaki Prefecture (24942-39-3) and Miyazaki Superintendent of Education (#0850-1713).

### Sample collections and DNA extraction

Blood samples are collected as a part of a going long term observations of the Japanese murrelet on Biroujima Island in Miyazaki Prefecture in western of Japan. Samples, consisting of a drop of blood, were collected from wild Japanese murrelet birds captured during fieldwork; this fieldwork was performed with the permission of the Ministry of the Environment, Japan. Following clotting of the blood within the needle (observed as a change of color), the clots were stored directly in ethanol solutions at room temperature. The sex of the birds was not known at collection. After a small amount of each blood clot was spun down and dried, DNA was extracted from the blood samples using the DNeasy Blood & Tissue kit (Qiagen) according to the manufacturer’s instructions for animal tissue. As a practical example to evaluate the new primer set, the DNA samples of Japanese quail were used. The feathers from the each Japanese quail were known the sex from morphological feature. The adult females of Japanese quail have pale colored breast feathers with the black colored speckles. On the other hand, adult males have plain rust-red feathers on the breast. DNA extraction was carried out from the feathers of the each male and female of Japanese quail using the DNeasy Blood & Tissue kit (Qiagen). In the course of the field work, one Japanese murrelet was found dead. Molecular sexing was carried out using a DNA sample extracted from feathers recovered from the carcass. The dead bird also was subjected to sexing by necropsy dissection of the reproductive organs.

### Preparation of primers

Four primer sets were prepared for PCR amplification from *CHD1Z* and *CHD1W* genes. Three primer sets, CHD1F/CHD1R^9^, 2250F/2718R^7^, and P2/P8^8^ were as reported previously; these pairs represent the most popular primer sets utilized by researchers all over the world for many species of birds^12^. The sequences of the oligonucleotides used and each annealing temperature are presented in Supplementary Table 1. In addition to the three primer sets, a newly designed primer set, CHD1-F1(Fwd; 5′-ATGGAAATGAGTGCATTGCAGAAG-3′)/CHD1-R1 (Rev;5′-CTTTTGAACCTTTACTCAGGGCTT-3′), was prepared for sexing the Japanese murrelet; these primers were designed based on the DNA sequences of the *CHD1Z* and *CHD1W* genes of chicken (Z:NM_204941.1, W:AF181826) and zebra finch (Z:AY217131, W:AY217129). Based on alignment with the chicken *CHD1Z* and *CHD1W* genes, the region spanned by the newly designated primer set is expected to correspond to intron 15 along with parts of the flanking exons 15 and 16. Using the prepared primer set, the PCR products amplified both from the introns of CHD1 genes in Japanese murelet were expected to be smaller than 300 bp.

### PCR conditions and analysis

For molecular sexing in the Japanese murrelet, PCR amplification was performed using the Accuprime^TM^*Taq* DNA Polymerase System (Invitrogen, Thermo Fisher Scientific). Amplification was carried out in a 10-ms carried out in a 10(Invitrogen mM Tris-HCl(pH 8.4), 1.5 mM MgCl_2_, 50 mM KCl, 0.2 mM each of dNTPs, 0.5 mM each of primers, and Accuprime^TM^Taq DNA polymerase. After an initial denaturation for 2 min at 94 °C, a 3-step PCR program was carried out as 35 cycles of 20 sec at 94 °C (denaturation), 30 sec at the respective primer-specific annealing temperature (annealing), and 40 sec. at 68 °C (extension). The PCR program was followed by a final extension of 7 min at 68 °C and a cooling phase at 4 °C. Amplified PCR products were confirmed by electrophoresis on 2% agarose gels or 5–20% polyacrylamide gels followed by ethidium bromide staining and visualization by UV transilluminator.

### Cloning of PCR products and DNA sequence analysis

To permit analysis of the DNA sequences of PCR products from the *CHD1Z* gene (on chromosome Z) and *CHD1W* gene (on chromosome W), PCR products for the *CHD* genes of Japanese murrelet were cloned into a plasmid vector using the PCR4-TOPO-TA cloning kit (Thermo Fisher Scientific). Nucleotide sequences were determined by Sanger sequencing.

### Accession codes

Three DNA sequences of CHD1-related PCR products including the region corresponding to the parts of two exons, exon 15 and exon 16, and the intron between the exons in *Synthliboramphus wumizusume* have been deposited in the DDBJ/EMBL/GenBank under accesstion numbers MN519214 from CHD1Z gene and MN519215 from CHD1W gene.

## Supplementary information

Supplementary dateset.
