# Supplementary material for: Molecular sexing in Japanese murrelet (Synthliboramphus wumizusume) and a tandem-repeat polymorphism on the W chromosome
Source: Sci Rep. 2020 May 22;10:8576. doi: 10.1038/s41598-020-65206-7 (PMC7244538; doi:10.1038/s41598-020-65206-7)
Supplement: Supplementary file 1 — Supplementary dateset. [file 41598_2020_65206_MOESM1_ESM.pdf]

Supplementary information for

Molecular sexing in Japanese murrelet (*Synthliboramphus wumizusume*) and a tandem-repeat polymorphism on the W chromosome

Hitoshi Hatakeyama, Yutaka Nakamura, Takahiro Konaka, Shin Nishida, Wannapimol Kriangwanich, Kazuyoshi Uematsu, Shuichi Tsuchida

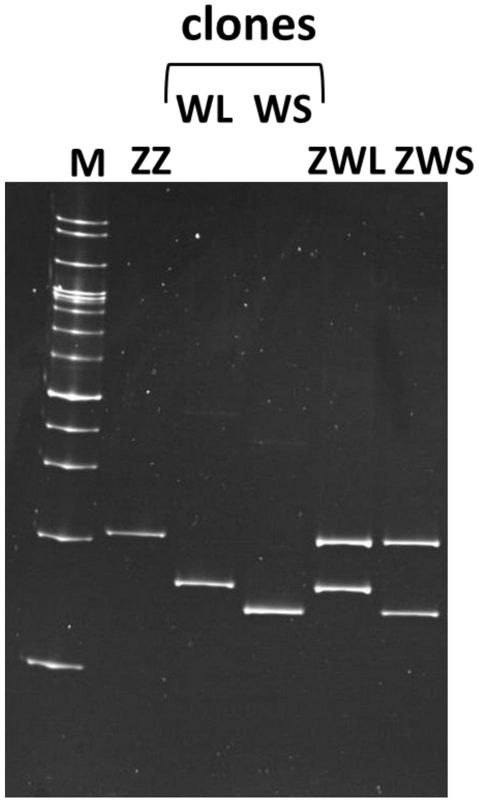

Suppelementary Figure 1. Polyacrylamide gel electrophoretic patterns of PCR products for sex identification.

Genotypes of each sample was indicated upper side in each lane. The pattern of ZZ is observed in male and ZW patterns were observed in female. Thereis a polymorphism in size in female product patterns, W-long(WL) and W-short(WS). The clones of polymorphic W fragments are evaluated on the gel after PCR amplification of cloned WL and WS, respectively. The M line indicates the 200-bp DNA ladder for a marker DNA.

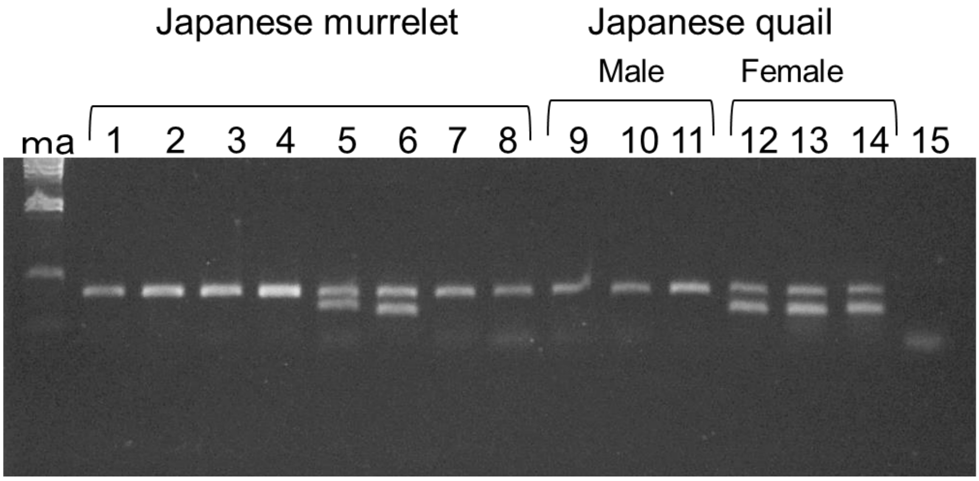

Supplementary Figure 2. Confirmation of the usefulness of the new primer set for molecular sexing. Confirmation of the usefulness of the newly designated primer set, CHD1F1F/FR1, for molecular sexing, using sexual two morphological bird, Japanese quail, as the samples of other species bird. To confirm the usefulness of the species-specific primer set, CHD1F1/CHD1R1, made for sexing of Japanese murrelet, the primer set was applied to DNA samples for molecular sexing of Japanese quail which sexes were identified from external morphological feature. Between the sexes identified from morphological features and from molecular DNA tests, no discrepancy was detected. The male in Japanese quail shows the simple pattern which comprised of one band from chromosome Z, and the female shows two bands. The upper bands were same as the band in male and smaller band from chromosome W. Japanese murrelet are also estimated to be male for lane No. 1- 4, 7 and female for 5 and 6. Lane 15 has no sample for negative control. Comparison of the patterns on agarose gel for identifying the sex between lane 5 and 6 reveals that both of PCR products shows to be female of Japanese murrelet, but there is difference in the migration of the PCR product. Product W in lane 5 is appeared to be longer than that in lane 6. Lane ma indicates the ramda phage DNA *Hind*III as a marker DNA.

Primer sets	Primer sequences(5′→3′)	Reference	Anneal Temp(℃)
CHD1F<br>CHD1R	TATCGTCAGTTTCCTTTTCAGGT<br>CTTTATTGATCCATCAAGCCT	Lee JC<br>(2010)	57
2250F<br>2718R	CTTACTGATTCGTCTAGGAGA<br>ATTGAAATGATCCAGTGCTTG	Fridolfsson AK<br>(1990)	52
P2<br>P8	TCTGCATCGCTAAATCCTTT<br>CTCCCAAGGATGAGRAAYTG	Grifiths R<br>(1998)	52
CHD1-F1<br>CHD1-R1	ATGGAAATGAGTGCATTGCAGAAG<br>CTTTTGAACCTTTACTCAGGGCTT	the present<br>study	55

Supplementary Table 1. The sequences of four primer sets for the PCR amplification for molecular sexing used in this study.
